# Hair Germ Model In Vitro via Human Postnatal Keratinocyte-Dermal Papilla Interactions: Impact of Hyaluronic Acid

**DOI:** 10.1155/2017/9271869

**Published:** 2017-10-10

**Authors:** Ekaterina Kalabusheva, Vasily Terskikh, Ekaterina Vorotelyak

**Affiliations:** ^1^Laboratory of Cell Biology, N.K. Koltzov Institute of Developmental Biology, 26 Vavilov St., Moscow 119334, Russia; ^2^Department of Regenerative Medicine, Institute of Translational Medicine, Pirogov Russian National Research Medical University, 1 Ostrovitianov St., Moscow 117997, Russia; ^3^Department of Cell Biology and Histology, Lomonosov Moscow State University, 1 Leninskiye Gory, Moscow 119234, Russia

## Abstract

Hair follicle (HF) reconstruction *in vitro* is a promising field in alopecia treatment and human HF development research. Here, we combined postnatal human dermal papilla (DP) cells and skin epidermal keratinocytes (KCs) in a hanging drop culture to develop an artificial HF germ. The method is based on DP cell hair-inducing properties and KC self-organization. We evaluated two protocols of aggregate assembling. Mixed HF germ-like structures demonstrated the initiation of epithelial-mesenchymal interaction, including WNT pathway activation and expression of follicular markers. We analyzed the influence of possible DP cell niche components including soluble factors and extracellular matrix (ECM) molecules in the process of the organoid assembling and growth. Our results demonstrated that soluble factors had little impact on HF germ generation and Ki67^+^ cell score inside the organoids although BMP6 and VD3 maintained effectively the DP identity in the monolayer culture. Aggrecan, biglycan, fibronectin, and hyaluronic acid (HA) significantly stimulated cell proliferation in DP cell monolayer culture without any effect on DP cell identity. Most of ECM compounds prevented the formation of cell aggregates while HA promoted the formation of larger organoids. In conclusion, our model could be suitable to study cell-cell and cell-niche interactions during HF reconstruction *in vitro*.

## 1. Introduction

Hair loss or alopecia is a widespread pathology; men and women at any age could be affected by any of the different types of alopecia. Several alopecia variants induce hair follicle (HF) miniaturization while others are associated with the complete loss of HF. Up to date, possible treatments include therapeutic agents (minoxidil, laser therapy, etc.) and surgical methods, including HF transplantation. One of the promising directions in HF research and treatment is reconstructing HFs from autologous cell source.

The growth and cycling activities of the HF are largely controlled by groups of epithelial and mesenchymal stem cells [1–3]. Epithelial stem cells give rise to progenitors which are the building blocks for HF morphogenesis during the hair cycle [4, 5]. Mesenchymal stem cells of HF, located in a structure called dermal papilla (DP), have a unique ability to direct skin interfollicular keratinocytes (KCs) to the follicular lineage [6, 7].

DP cells maintain their inducing properties for several passages in culture, although the loss of contextual and positional cues from DP niche results in a rapid decline of their abilities that also involves the downregulation of specific markers [8–13]. DP niche components include at least soluble factors and extracellular matrix (ECM) molecules. Components of WNT and BMP signaling pathways are among the main players during the HF development and cycling. It was shown that HF germ keratinocytes express these molecules for the regulation of HF cycle [14]. These factors support and stimulate hair-inducing abilities of DP cells *in vitro* [10–13]. Besides hair-inducing abilities, the key feature of DP cells is a tendency to aggregate in culture reproducing the initial steps of HF formation. This process depends on ECM components, especially DP-specific protein versican [15]. Human HF development has not been well investigated, but basing on evidences obtained from rodent research, soluble factors mentioned above and other molecules are involved in the process of DP cell condensation. DP niche could be reconstructed using KCs to provide all the necessary soluble factors and 3D culturing to stimulate DP aggregation processes. DP cells in spheroid cultures partially restore the inductive capabilities as has been demonstrated in the recent research [9, 10]. Combining DP cells and KCs in 3D environment resulted in the reconstruction of several essential epithelial-mesenchymal interactions typical for human HF [16, 17], although the optimal conditions allowing the complete HF follicle development from postnatal cells are still not being found.

Here, we developed two cultured HF germ models: coated and mixed aggregates. We evaluated the influence of DP cell identity on HF germ formation and expression of HF markers. Mixed aggregates appeared to be the most promising model. Lef1 expression confirmed WNT pathway activation. KCs switched to HF differentiation lineage demonstrating P cadherin expression. DP hair-inducing abilities correlated with the size and dividing cell ratio inside the aggregate. Soluble factors (BMP6, VD3, and VPA) maintained DP cell identity, while several ECM components (aggrecan, biglycan, fibronectin, and hyaluronic acid (HA)) significantly stimulated cell proliferation in 2D cultures. Nevertheless, only HA induced significant upregulation of the proliferation and increased the size of aggregates. Our results may provide the new *in vitro* method of HF development, and the model could be suitable to study cell-cell and cell-niche interactions during HF reconstruction *in vitro*.

## 2. Materials and Methods

### 2.1. Primary Cell Cultures

Human scalp biopsies were obtained after face-lift surgery from informed and consented patients aged 46 to 60 years from The Clinic of Active Longevity, Institute of Beauty on Arbat. Prior to cell isolation, skin samples were washed with Hank's solution (PanEko) with gentamicin.

DP cells were isolated by the technique developed by Wu et al. [18] and modified by Chermnykh et al. [19]. Briefly, the skin was incubated in 0.5% dispase (Gibco) at 4°C overnight. Subcutaneous fat was separated manually by surgical scissors and incubated in 0.2% collagenase type I (Gibco) for 2-3 h at 37°C. After this step, HF bulbs were separated from fat by pipetting and centrifuging. To obtain DPs, HF bulbs were additionally incubated in 0.2% collagenase type I for 3-4 h at 37°C. DPs were purified by a series of low speed centrifuge. The cells were cultured in AmnioMAX™-II medium (Gibco). Cells at passages 1–4 were used for all experiments if another one is not indicated.

The human lung fibroblasts (LF) were purchased from ATCC (ATCC® CCL-204™) and cultured in AmnioMAX-II medium.

For KC isolation, the epidermal layer was separated from the skin by incubation in dispase at 4°C overnight. The epidermal sheet was disrupted by trypsinization for 15 min. KC suspension was inoculated in DMEM/F12 medium (PanEko) containing 4 mM glutamine (Gibco), 10% fetal bovine serum (FBS) (HyClone), 10 ng/ml EGF (Sigma Aldrich), 5 mg/ml insulin (Sigma Aldrich), and 0.25 mg/ml isoproterenol (Sigma Aldrich).

Overall, DP cultures obtained from 17 donors were used. Each DP culture was combined with 3–5 different donors of keratinocytes. Overall, 26 donors of keratinocytes were used. Each experiment was performed using DP from, at least, three donors. Results were compared in each pair of donors separately meaning that the control and the experiment group in each case were from the same donors of DP and keratinocytes. Results were considered to be valuable if they were reproduced in three DP cultures from different donors. All the cells obtained from KC and DP cultures were deposited in the Cell Culture Collection of the Institute of Developmental Biology, RAS.

### 2.2. Soluble Factors and ECM Molecules

The following soluble factors at designated concentrations were used in the study: bone morphogenetic factor 6 (BMP6, R&D systems, 100 ng/ml), 1*α*,25-dihydroxyvitamin D3 (VD3, Sigma Aldrich, 100 nM), valproic acid (VPA, Sigma Aldrich, 2 mM), Wnt3a (R&D systems, 100 ng/ml), Wnt5a (R&D systems, 100 ng/ml), and Dickkopf1 (Dkk1, R&D systems, 100 ng/ml).

Among ECM components, we used aggrecan, biglycan, fibronectin, matrigel, collagen type I, hyaluronic acid (HA), and chondroitin sulphate A. Multiwell plates were coated with aggrecan (R&D systems) at 0.4 nmol/cm^2^, biglycan (R&D systems) at 0.2 nmol/cm^2^, fibronectin (R&D systems) at 0.4 nmol/cm^2^, chondroitin sulphate A (CS) (Sigma Aldrich) at 0.05 mg/cm^2^, or 0.05 mg/cm^2^ HA (Abcam, MW 1.6 kDa). Type I collagen solution extracted from the rat tail tendons was incubated at 37°C for 30 min. Matrigel (BD Biosciences) was diluted at a concentration 0.12 mg/cm^2^ and incubated at 4°С overnight.

### 2.3. Lentiviral Transduction

Lentiviral constructions bearing red fluorescent protein *TagRFP* gene were provided by the Eurogene Company (Russia). DP cells at passage one and LF at passage 3 were transfected in serum-free AmnioMAX-II medium with the addition of polybrene (Sigma Aldrich) with tenfold excess concentration of viral particles.

### 2.4. DP, LF, and KC Coculture

For monolayer culture, DP cells, labeled by RFP, and KCs were trypsinized, mixed in 1 : 1 proportion, seeded in a 48-well plate (Corning) at a concentration of 10^5^ cells per well in DMEM medium (PanEko) containing 4 mM glutamine and 10% FBS, and cultured for three days.

To obtain mixed aggregates, DP cells or LF, labeled by RFP, and KCs were mixed in 1 : 1 proportion and cultured in a hanging drop in DMEM medium containing 4 mM glutamine and 10% FBS at a concentration of 7 × 10^3^ cells per aggregate. Cells were cultured for 3 to 14 days. To assess the influence of soluble factors and ECM molecules on aggregate generation, BMP6, VD3, VPA, Wnt3a, Wnt5a, Dkk1, aggrecan (0.4 *μ*M), biglycan (0.2 *μ*M), fibronectin (0.4 *μ*M), CS (0.5 mg/ml), 1.12 mg/ml matrigel and collagen I at a dilution 1 : 10, and 0.5 mg/ml HA were added to cell suspension prior to the cell aggregation.

To obtain coated aggregates, DP cell suspension, labeled by RFP, was placed in a hanging drop at a concentration of 3.5 × 10^3^ cells per aggregate in DMEM medium containing 4 mM glutamine and 10% FBS. After three days in culture, aggregates were transferred to preliminarily prepared hanging drops containing 3.5 × 10^3^ keratinocytes and then additionally cultured for 3 to 14 days.

### 2.5. Alkaline Phosphatase Staining

DP cells were fixed in 70% ethanol for 15 min at room temperature and then washed with phosphate saline buffer (PBS) pH 7.5 (Gibco). Cultures were incubated in NBT/BCIP solution (Roche) for 30 min at room temperature protected from light. Samples were analyzed using an Olympus IX51 fluorescent microscope equipped with an Olympus DP70 camera.

### 2.6. Immunocytochemistry

The monolayer cultures were fixed in 4% paraformaldehyde (PFA) (Riedel-de Haen) for 15 min at room temperature and then washed with phosphate saline buffer (PBS) pH 7.5 (Gibco). Primary antibodies anti-Ki67 (Abcam, ab16667) and anti-versican (R&D systems, AF3054) were diluted in 1 : 50 and 1 : 20, respectively, in permeabilization buffer consisting of PBS, with addition of 1% Triton X-100 (AppliChem), 1% Tween 20 (AppliChem), 0.5% bovine serum albumin (Sigma Aldrich), and sodium azide (Sigma Aldrich). Samples were incubated at 4°C overnight and then washed with PBS for one hour. Secondary anti-rabbit IgG Alexa fluor 488 antibodies (Life technologies, A21206) and anti-goat IgG Alexa Fluor-488 (Life technologies, A21467) were diluted in PBS in 1 : 500 and incubated with the samples for 1 h at room temperature. DAPI (Biotium) was used for nucleus staining at a concentration of 1 mg/ml. Samples were analyzed using a fluorescent microscope (IX51, Olympus) equipped with a DP70 camera.

Aggregates were fixed in 4% PFA for 45 min at room temperature and incubated in permeabilization buffer for 1 h. The following primary antibodies were used: anti-Ki67, anti-Lef1 (Santa Cruz, sc-8591) diluted in 1 : 200, anti-P cadherin (R&D, AF960) diluted in 1 : 20, and anti-EpCam (R&D, MAB861) diluted in 1 : 15. Then, samples were processed as indicated above. Toto-3 (Life technologies) was used for nucleus staining at a dilution of 1 : 300 in Tris-HCl buffer (Helicon) with RNase (Fermentas) pretreatment, or alternatively, DRAQ5 (Life technologies) was used at a dilution of 1 : 1000 in PBS. Samples were mounted in fructose and analyzed using a confocal microscope (Leika SP5, Germany).

### 2.7. Gene Expression Analysis Using the qRT-PCR Method

RT-qPCR was used to quantify the mRNA expression of ALP, Versican, WNT5a, LEF1, *β* catenin, P cadherin, EpCAM, TCHH, Keratin 75, Keratin 35, and Keratin 32. RNA was isolated from each group by using RNAzol reagent (MRC) according to the manufacturer's instructions. First-strand cDNA was synthesized using a QuantiTect Reverse Transcription Kit (Qiagen) according to the manufacturer's instructions. qPCR was performed in triplicate using a CFX96 Real-Time PCR system (Bio-Rad Laboratories) under the following conditions: 10 min at 95°C, followed by 45 cycles of 15 sec at 95°C, and 1 min at 60°C for qPCR amplification. The reaction was performed in a total volume of 25 *μ*l, containing 5 *μ*l SYBR Low Rox Master Mix (Eurogen), 1 *μ*l cDNA, 0.5 *μ*l each primer, and 18 *μ*l sterile distilled water. Target gene concentration was calculated using calibration curve and normalized to the level of HPRT. The primers used are indicated in Table 1.

### 2.8. Imaging, Analyzing, and Statistics

The fluorescent images of the monolayer cultures were analyzed automatically to define the ratio of actively proliferating cells depending on the ECM or the soluble factor used with the particular culture. The assay was carried out using the CellProfiler version 2.2.0 software. The share of proliferating cells in a monolayer was identified as the ratio of Ki67-positive cell nuclei to the total number of cell nuclei in the picture. From 30 to 50, images of each sample taken with 20x magnification were used for this analysis.

To evaluate the proliferation ratio in mixed aggregates, nuclei were counted on z stacks from confocal imaging using ImageJ.

Statistica 8 was used for the statistical analysis. Data are shown as a mean ± standard error of the mean and represent the average of three separate experiments. The Mann–Whitney *U* test was used to determine the statistical significance of observed differences at the level of *p* ≤ 0.05.

## 3. Results and Discussion

### 3.1. General Characteristics of the DP-KC Aggregates

**Figure 1:**
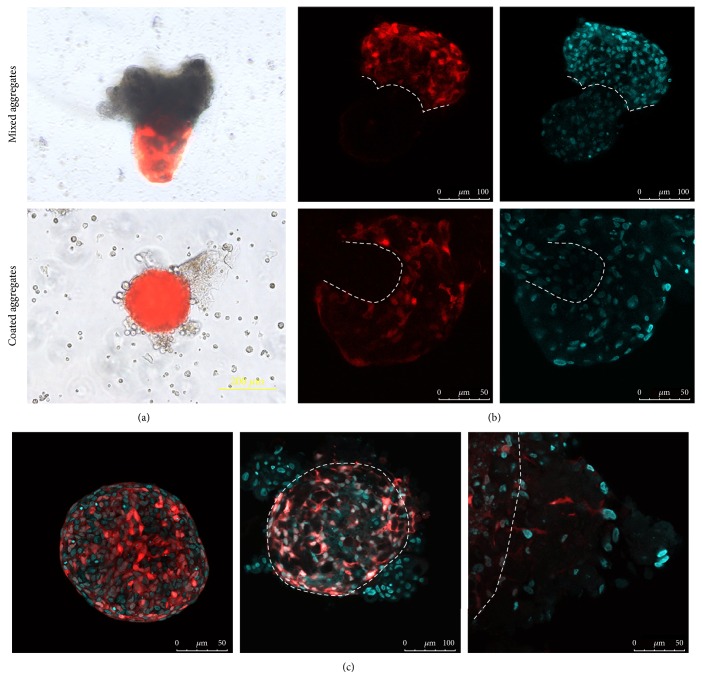
Aggregate morphology. (a) Mixed and coated aggregates. Fluorescent and light microscopy. Scale bar 200 *μ*m. (b) Mixed aggregate structure. Confocal microscopy. Scale bar 100 *μ*m for upper row; scale bar 50 *μ*m for bottom row. (c) Coated aggregate morphology. Left image is DP spheroid before coating, and middle and right images are coated aggregates. Dashed lines show the borderline between DP aggregate and KCs. Confocal microscopy. Scale bar 50 *μ*m for left and right image and scale bar 100 *μ*m for middle image. DP cells are labelled with RFP. Nuclei are counterstained with TOTO3 (cyan color). Dashed lines show the borderline between DP cells and KCs.

**Figure 2:**
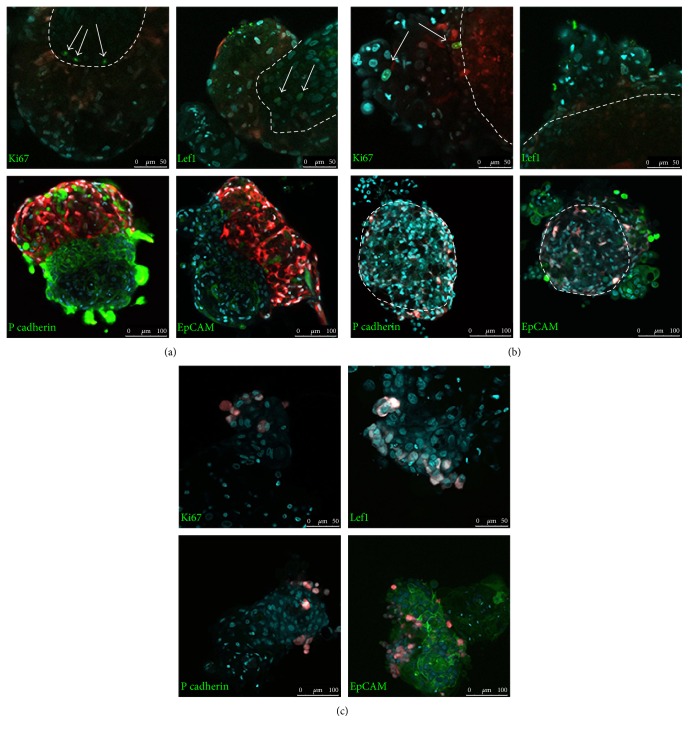
Cell proliferation and HF marker expression in aggregates: (a) mixed DP-KC aggregates; (b) coated DP-KC aggregates; and (c) LF-KC mixed aggregates as a control. Confocal microscopy. DP cells and LF are labelled with RFP. Immunocytochemistry for Ki67, P cadherin, Lef1, and EpCAM as indicated (green). White arrows point to Lef1 and Ki67 positive nuclei; white dotted lines show the borderline between DP cells and KCs. Nuclei are counterstained with TOTO3 or DRAQ5 (cyan color). Scale bar 50 *μ*m for Ki67 and Lef1 staining and 100 *μ*m for P cadherin and EpCAM staining. Dashed lines show the borderline between initial DP aggregate and KCs.

In mixed aggregates (prepared as it is designated in Materials and Methods), spontaneous cell sorting occurred. DP cells interacted with each other and produced spheroid structures while KCs tended to form extended trabeculae. The length of trabeculae varied among donors. These epithelial structures were somewhat similar to the tubules grown in the collagen gel in the presence of DP or conditioned medium [19]. In the course of aggregate preparation, KC trabeculae embedded into DP spheroids: several trabeculae might be embedded into one DP spheroid, while one trabecula could bear several DP aggregates. In rare cases, DP cells made a belt surrounding KC trabeculae (Figure 1(a)). The borderline between DP cells and KCs could be well distinguished. We did not observe any DP cells penetrating KC trabeculae. Single KCs were found inside DP cell aggregates (Figure 1(b)). Proliferating Ki67^+^ KCs were found exclusively at the borderline between DP cells and KCs (Figure 2(a)). KCs maintained proliferation at least for 5 days. Proliferative DP cells were detected throughout the aggregates. We analyzed the expression of early HF marker Lef1 and found that proliferative KCs at the site of epithelial-mesenchymal contact were positive for this marker (Figure 2(a)). Lef1 expression in DP cells was irregular and was not restricted to any specific zone (data not shown).

To obtain coated aggregates, DP cell spheroids were transferred into suspension of KCs, which then attached to the spheroid surface (Figures 1(a) and 1(c)). We noticed that DP cells formed processes and sometimes migrated into the KC layers that resulted in the border between DP spheroid, and KCs became indistinct (Figure 1(c)).

We did not identify any Ki67^+^ nuclei among KCs in coated aggregates. DP cells behaved differently: they proliferated and migrated through the KC layers (Figure 2(b)). In coated aggregates, KCs and DP cells were negative for Lef1 (Figure 2(b)). The system of coated aggregates did not support epidermal proliferation: KCs attached to the DP spheroid surface completely exfoliated after 5–10 days.

Lef1 expression and KC proliferation indicated that mixed aggregates demonstrated epithelial-mesenchymal interaction, similar in some aspects to that in HFs. We analyzed aggregates for the markers of human HF placode P cadherin and EpCAM [20] and found their expression in KCs inside mixed aggregates (Figure 2(a)). We did not observe P cadherin expression in coated aggregates, while KCs were positive for EpCam (Figure 2(b)). P cadherin expression indicates the onset of neofolliculogenesis, since this protein is expressed not only during the development of HFs but also in HF cycling [21]. P cadherin expression was observed in regenerated HFs induced by DP cell transplantation [22]. EpCam was found to be expressed in early HF placodes of 11-12 week human fetus and seems to be the sign of the earliest steps of HF morphogenesis [20].

Both types of aggregates were cultured up to 14 days; however, we did not observe any changes in morphology indicating further development of HFs. Basing on aggregate characteristics, we discarded coated aggregates from our further study.

We confirmed the specificity of HF marker expression using LF instead of DP cells in mixed aggregates. LF were chosen to avoid the possible contamination of skin fibroblast culture with DP cells and to avoid the nonspecific expression of markers. Mixed aggregates with LF did not correspond to those with DP cells: fibroblasts attached as small clusters on the surface of KC spheroids (Figure 1(c)). Neither KCs nor LF were positive for Lef1 or Ki67. P cadherin expression was absent while EpCam expression was observed (Figure 2(c)). The results provide evidence that the process of HF morphogenesis in mixed aggregates is induced by DP cells, and Lef1 and P cadherin are the specific markers of an onset of this process. In our hands, EpCAM staining could not be used as a reliable marker of folliculogenesis as weak staining for this marker was found in control experiments with LF and even in monolayer KCs (data not shown).

DP cells and KC suspension are able to self-organize and form mature HF after subcutaneous cotransplantation into immunodeficient mice [23, 24]. Fetal cells have a potential to reaggregate and develop to the HF-like structures which are suitable for transplantations [25–27], while adult cells lose these abilities. Similarly, the use of embryonic cell and bioprinting technologies allows the establishment of tooth primordia which are capable to replace adult teeth [28, 29], although postnatal cells are ineffective in tooth regeneration [30]. Here, we demonstrate that postnatal cells are able to self-organize and to reproduce initial steps to form hair germ-like structures.

The inability of coated aggregates to reproduce HF epithelial-mesenchymal interactions highlights the significance of self-organization processes which take place during the arrangement of mixed aggregates. Epithelial cells from other organs have been demonstrated to exhibit high self-organization abilities under 3D conditions [31–33]. Our results have revealed that the presence of DP cells is sufficient for proper KC self-organization in hanging drops when used as a single-cell suspension. Yen and coauthors [34] have obtained HF organoids using low-adhesive conditions. A structure of these organoids was similar to our coated aggregates; nevertheless, key features of folliculogenesis onset were observed which is in contradiction with our results. These aggregates were obtained using cell culture conditions, which supported the self-aggregation and self-organization. In other words, cells managed the process of spheroid formation by themselves as well as in our mixed aggregates; the difference in final organoid structure is explained by different forces which stimulated the process of aggregation: gravitation or specified adhesiveness.

### 3.2. Expression of Specific Markers of DP Cells and Folliculogenesis in Different Types of DP-KC Aggregates

**Figure 3:**
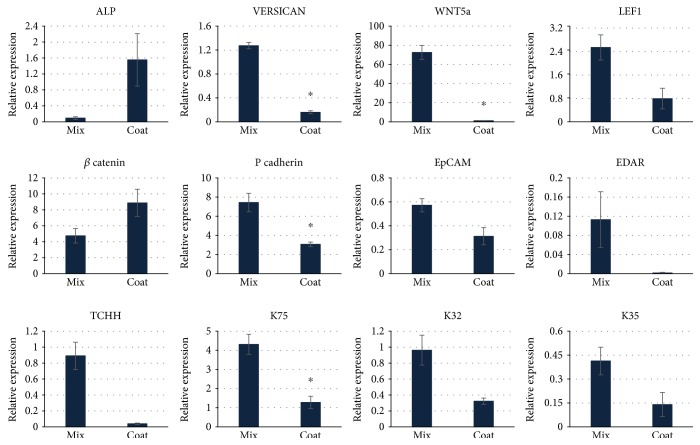
Relative expression of DP and folliculogenesis markers studied with RT-qPCR. Expression level of each gene was normalized to HPRT. ^∗^*p* < 0.05, Mann–Whitney *U* test.

We further evaluated the difference between mixed and coated aggregates by analyzing the gene expression of DP and HF epithelial markers (Figure 3). Mixed aggregates showed increased expression of such DP markers as *versican* and *Wnt5a* while coated ones demonstrated a high level of *alkaline phosphatase* (ALP). An elevated level of *Lef1* in mixed aggregates confirmed the initiation of folliculogenesis while increased *β catenin* expression was found in coated aggregates. Several markers of folliculogenesis including *P cadherin* and *K75* were upregulated in mixed aggregates. These results confirm the onset of morphogenetic processes in mixed aggregates. The increase in the expression of DP markers in coated aggregates may be accounted for the extended period of DP cell cultivation in this case as preliminary prepared DP aggregates have been used in this variant of aggregates. Prolonged cultivation of DP cells in spheres upregulates the expression of specific DP genes.

### 3.3. Monolayer Modeling of DP-KC Interaction

**Figure 4:**
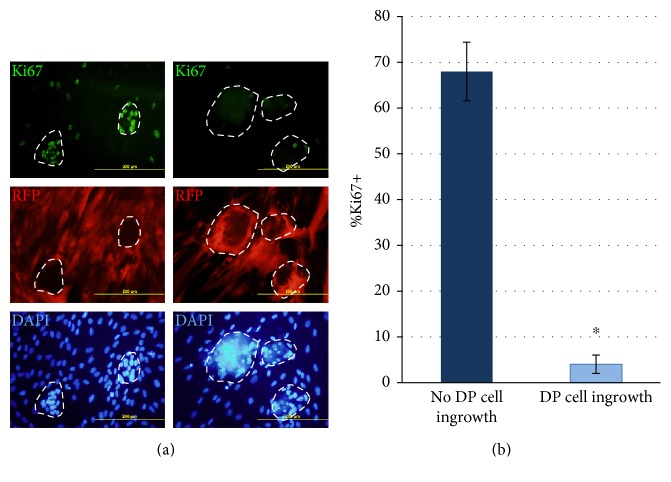
(a) KC proliferation in monolayer coculture with (right column) and without (left column) the contact/ingrowth of DP cells. DP cells are labelled with RFP. Nuclei are counterstained with DAPI. Scale bar is 200 *μ*m. Fluorescent microscopy. (b) KC proliferation rate in clumps depending on DP cell contact/ingrowth. ^∗^*p* < 0.05, Mann–Whitney *U* test.

To clarify the interaction between two cell types within aggregates, we imitated the interaction between DP cells and KCs in aggregates with monolayered coculture. We mixed DP cells and KC suspension and cultured it on the adhesive surface. After 3 days, DP cells formed confluent monolayer, keratinocytes adhered as separate clumps, several clumps resided atop of the DP cells monolayer, and others were located on the plastic and surrounded by DP cells (Figure 4(a)).

We observed high proliferation level in the KC clumps located on the bottom of a culture dish. KC clumps atop of DP cells comprised few Ki67^+^ cells. We also observed DP cells, which had contacted with these clumps and formed processes inside KC sheet. The contact with DP cells appeared to reduce the number of Ki67^+^ KCs more than 10 times (Figure 4(b)). The results demonstrate that close contact of DP cells with KC layers inhibits the proliferation of KCs. It corresponds to the absence of KC proliferation in coated aggregates. Thus, this was not exclusively the attribute of 3D culture, and the pattern of mutual influence between DP cells and KCs in coated aggregates is similar to that in the monolayer culture reflecting some basic mode of interaction. Noteworthy, DP represents a highly adhesive structure comprising tightly bounded cells, which rarely escape DP. The only way of migration in natural niche is migration into connective tissue sheath. In this situation, KCs of the matrix never contact DP cells. In case of skin and HF damage, rapid migration of DP cells may cause KC elimination from the damaged site.

### 3.4. DP Cell Identity Influences the Size of Aggregates and Cell Proliferation

DP cells are distinguishable from other skin cell types by their ability to induce HF formation after transplantation into the skin [6, 7]. *In vivo* DP cells interact with HF KCs, and these interactions promote HF cycle [1–3]. In culture, DP cells lack their niche essential compounds that results in quick loss of hair-inducing abilities upon passaging [8–13].

**Figure 5:**
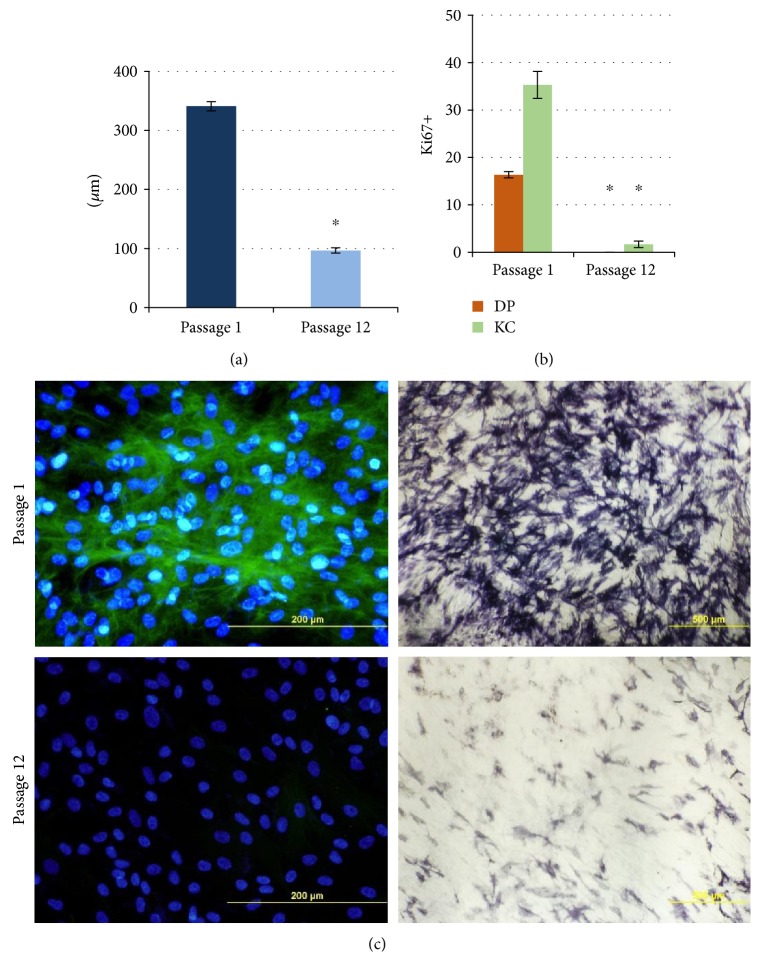
(a) The size of mixed aggregates with DP cells of passages 1 and 12. (b) The number of Ki67^+^ nuclei in DP cells and KCs in mixed aggregates per one aggregate generated with DP cells of passages 1 and 12. (c) Versican and ALP expression cultures of DP cells of passages 1 and 12. Versican: immunocytochemistry, fluorescent microscopy, scale bare 200 *μ*m. ALP: histochemistry, bright field microscopy, scale bare 500 *μ*m. ^∗^*p* < 0.05, Mann–Whitney *U* test.

We evaluated the impact of DP identity on hair germ generation using DP cells of passages one and 12. The decrease of hair-inducing abilities was manifested by the drop in ALP and versican expression (Figure 5(c)). Low DP hair-inducing abilities (passage 12) resulted in smaller aggregates. Aggregates containing DP cells from the first passage were three times bigger than those made of cells from passage 12 (Figure 5(a)). The reason for size diminishment was the inability of passage 12 DP cells to produce single aggregate per one hanging drop. Instead, they formed 5-6 separate small aggregates per one drop.

HF marker expression was not affected (data not shown) whereas the number of Ki67^+^ cells was dramatically decreased in aggregates with late passage DP cells (Figure 5(b)). Therefore, the downregulation of DP hair-inducing abilities resulted in inability to form aggregates properly and to stimulate KC proliferation.

### 3.5. Impact of Soluble Factors and ECM Components on DP Identity

Previous studies reported various approaches to maintain intrinsic characteristics in cultured DP cells, including the addition of certain soluble factors [10–13] and ECM compounds [35]. Since high hair-inducing abilities of DP cells are important for the aggregate formation and KC proliferation level maintaining, we tried to utilize this approach in order to improve the quality of DP cell monolayer culture before the generation of DP-KC aggregates and subsequently to facilitate aggregate acquisition.

Bioinformatics analysis revealed the involvement of Wnt, BMP, and FGF signaling pathways in the maintenance of human DP properties [10]. According to the above, to identify the best conditions for DP cell maintaining prior to inclusion into aggregates, we incubated them with the following soluble factors and molecules: BMP6, VD3, VPA, Wnt3a, and Wnt5a. We used Dkk1 as a negative control to compare with Wnts. FGF20 was included as the factor promoting DP cell aggregation during HF development [36].

**Figure 6:**
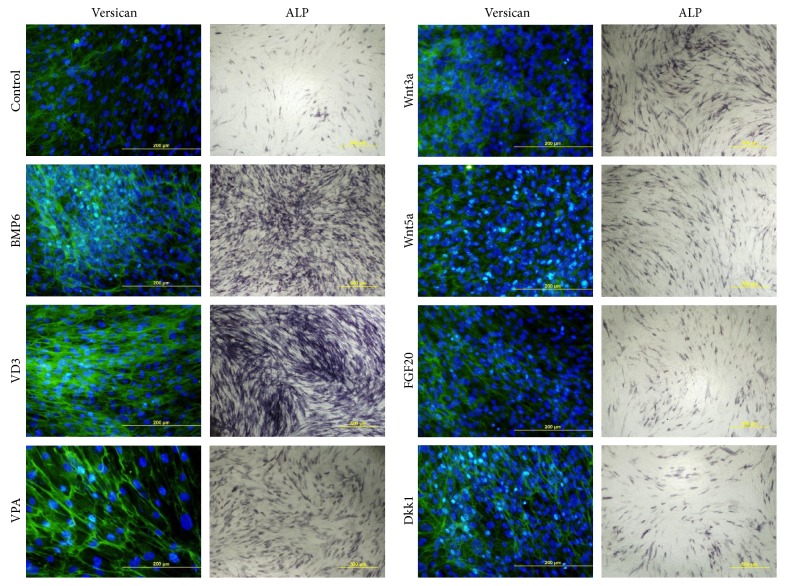
Versican and ALP expression in the presence of soluble factors. Versican: immunocytochemistry, fluorescent microscopy, scale bare 200 *μ*m. ALP: histochemistry, bright field microscopy, scale bare 500 *μ*m.

We analyzed the expression of DP-specific markers, namely, ALP and versican. Basing on immunocytochemical analysis, the most significant upregulation was recorded in cultures with the addition of BMP6, VD3, and VPA (Figure 6). Therefore, we suggested that these factors would be the most promising in terms of aggregate generation.

**Figure 7:**
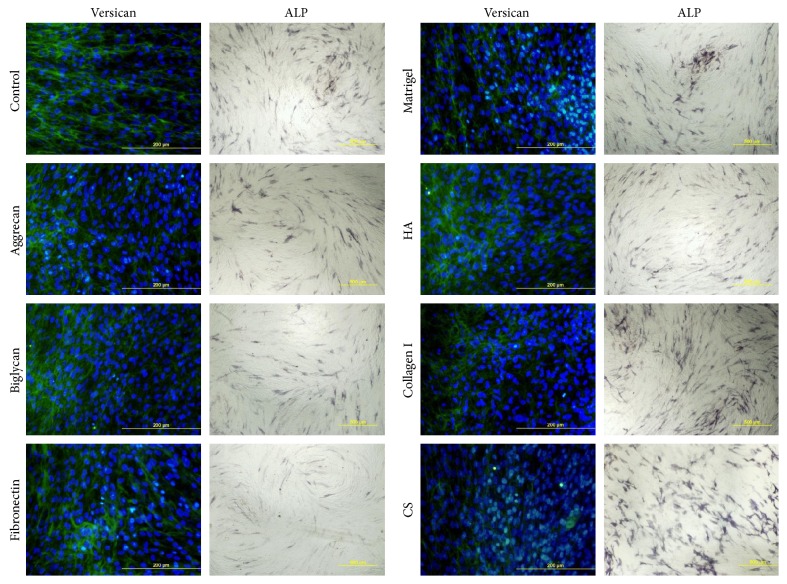
Versican and ALP expression in DP cells cultured on ECM compounds. Versican staining was analyzed using fluorescent microscopy; scale bare 200 *μ*m. ALP staining was analyzed using bright field microscopy; scale bare 500 *μ*m. HA: hyaluronic acid; CS: chondroitin sulphate.

Further, we evaluated the influence of proteoglycans (aggrecan, biglycan, and fibronectin), glycosaminoglycan CS, and collagen I on DP marker expression. Furthermore, we tested matrigel as a basal lamina substitute and HA, since it is widespread in the embryonic skin where multiple events of HF morphogenesis occur [37]. We did not observe the upregulation of ALP or versican expression in any cases (Figure 7()).

**Figure 8:**
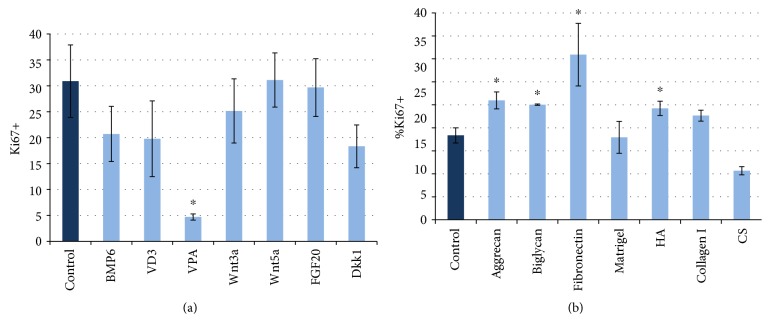
(a) The rate of Ki67^+^ nuclei in DP cell culture in the presence of soluble factors. (b) The rate of Ki67^+^ nuclei in DP cell culture in the presence of ECM components. ^∗^*p* < 0.05, Mann–Whitney *U* test. HA: hyaluronic acid; CS: chondroitin sulphate.

Since proliferation level is important for proper HF morphogenesis, we analyzed the influence of soluble factors and ECM molecules on the proliferation of DP cells in monolayer culture to select the most promising for aggregate generation. Soluble factors did not affect DP cell proliferation except VPA, which suppress cell division (Figure 8(a)). We found that proteoglycans and HA upregulated the rate of proliferative cells, while none of ECM components under investigation retarded proliferation (Figure 8(b)).

In native niche, DP cells are supported by soluble factors produced by epithelial stem cells from the bulge and hair germ regions. Both types of WNT pathways operate the HF development and cycling. VPA has been shown to be a prospective WNT signaling activator and stimulator of HF regrowth [38, 39]. BMP signaling molecules regulate the differentiation processes during the HF cycling and development [11]. Therefore, our results concerning the expression of DP markers are in agreement with previous studies. The absence of proliferation stimulation could be a result of a specific status of adult DP cells with high hair-inducing abilities [40].

DP cells produce a wide diversity of ECM compounds, including proteoglycans and collagens. The composition and contents are changed during the hair cycle; this suggests the pivotal role of ECM in the modulation of growth factor signaling and regulations of differentiation and proliferation processes [41–43]. It has been shown that canine DP cells upregulate the expression of Wnt/*β* catenin pathway members on laminin substrate [41].

Our results demonstrated that ECM molecules do not have an impact in human DP hair-inducing abilities recovery but influence cell proliferation. HA supports DP cell proliferation and allows the clonal growth of these cells in 3D environment [44]. It is also suitable for other mesenchymal cell types [45, 46]. *In vitro*, several ECM compounds regulate the processes of DP cell aggregation [47–49].

### 3.6. Impact of Soluble Factors and ECM Components on Aggregate Formation and Development

As DP cell-specific marker expression strongly correlates with their hair-inducing abilities, we suggested BMP6, VD3, and VPA to be promising agents for promoting aggregate development.

**Figure 9:**
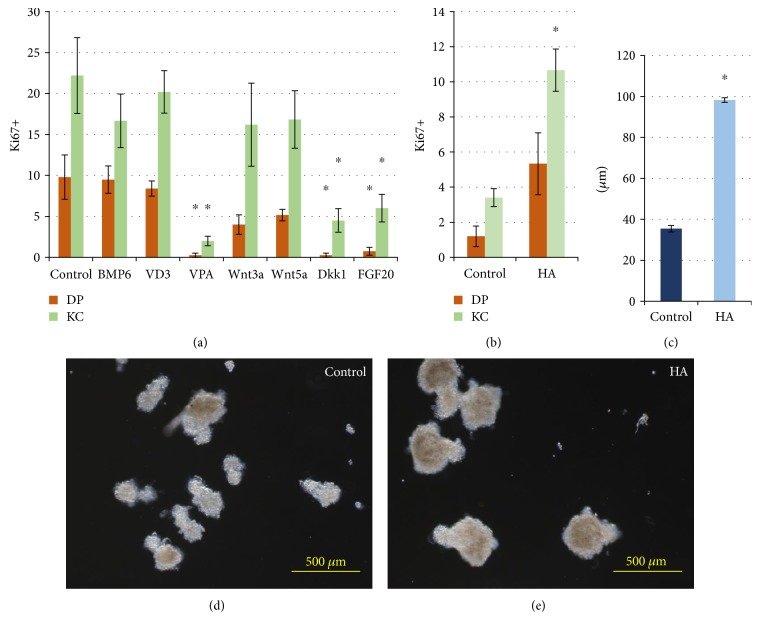
(a) The number of Ki67^+^ nuclei of DP cells and KCs in mixed aggregates per one aggregate, obtained in the presence of growth factors. (b) The number of Ki67^+^ nuclei of DP cells and KCs in mixed aggregates per one aggregate, obtained in the presence of HA. (c) The size of mixed aggregates in the presence of HA. The appearance of aggregate obtained (d) in control cultures and (e) in the presence of HA; bright field microscopy, ^∗^*p* < 0.05, Mann–Whitney *U* test.

All of the substances studied were added to cell suspension in the course of aggregate generation. In our hands, we did not observe any stimulation effect on cell proliferation inside aggregates neither from BMP6, VD3, and VPA nor from other substances. Moreover, VPA and FGF20 demonstrated the inhibitory effect (Figure 9(a)).

Further, we created mixed aggregates in the presence of ECM molecules previously tested in monolayer culture. Surprisingly, all the compounds prevented the aggregate formation except HA and chondroitin sulphate. HA addition resulted in the upregulation of KCs and DP cell proliferation (Figure 9(b)) and increased the size of aggregates (Figures 9(c), 9(d), and 9(e)) while addition of chondroitin sulphate produced no effect on the parameters of the aggregates (data not shown). According to our results in monolayer culture, VPA suppresses cell proliferation irrespectively of culture conditions, while FGF20 and Dkk1 decrease the number of Ki67-positive cells only in 3D culture with KCs. These revealed several key features about epithelial-mesenchymal interactions in mixed aggregates: stimulation of DP hair-inducing abilities is not enough to upregulate KC proliferation. The observations indicate that DP cells in the monolayer respond differently as compared to the same cells in mixed aggregates, where they are involved in epithelial-mesenchymal interactions. It could be suggested that 3D environment has an additional impact on DP cells themselves inducing the recovery of their intrinsic features as it was shown earlier [9]. It may be speculated that DP cells in 3D conditions have quite different properties as compared to those in the monolayer culture, and shifting them into aggregates has a prevailing effect on their behavior. Consequently, soluble factors did not produce an expected additional effect in terms of hair germ development. Significant inhibition of cell proliferation with Dkk1 indicates that Wnt activation is indispensable for proper aggregate arrangement and initial steps of *in vitro* folliculogenesis. Inefficiency of Wnt members to promote HF morphogenesis demonstrates that the Wnt pathway in aggregates is activated basically by intercellular contacts rather than paracrine signaling.

HA stimulates Wnt pathway activation in mesenchymal stem cells [45]. Apparently, this activation is carried out by cell contacts; this confirms our hypothesis about major role signaling via cell contacts rather than paracrine factors in aggregates. Therefore, HA may be further used for the establishment of proper cell-cell contacts and signaling in the course of HF *in vitro* development. Other ECM molecules did not facilitate aggregate formation that may indicate their secondary appearance inside native DP in the process of condensation in fetal skin.

## 4. Conclusion

In conclusion, the long-term goal of our investigation is to develop an easily obtainable model system that simulates human HF germ in culture, which could be useful for HF biology research and for clinical applications, providing the suitable system for drug testing and prospective field in HF reconstruction. The hair germ-like organoid developed in this study allows the search for niche factors maintaining HF morphogenesis.

## Figures and Tables

**Table 1:** Primers for real-time polymerase chain reaction.

Target	Forward primer	Reverse primer
hHPRT	ACCAGGTTATGACCTTGATT	AAGTTGGCCTAGTTTATGTT
ALP	GCACCTGCCTTACTAACTCC	AACATAGACACTGCCCTCATC
Versican	TCCAACAGGAAAGGAATGAA	TTACTGGGGACAGTGAGGTG
WNT5a	GGGTGATGCAGATAGGTAGG	TCAGGTGTAGGGACAGGAAT
LEF1	TGAAGGTGATTCTTGGGTTAT	CACGGGCACTTTATTTGACT
*β* Catenin	CAGCAGCAATTTGTGGTAGG	TAGCTCTTCAGGAAGACGGA
P cadherin	CACATCTGGGTTAAGGAGTT	CAGGAGAAGGCACAGTCGTA
EpCAM	CAGCGGTTCTTTTGGCATAC	TCCCCATTTACTGTCAGGTC
EDAR	TTGCCTCCTTTCTACTGTTGC	GCTTACCTTCCACGACTCCA
TCHH	CTCCTTGAAAGGGAATTTGG	TTCCTTGCTCTGGTCTCCTC
Keratin 75	TCAAAGTCAGGTAAGTGGGAGA	CAAGATGAAGGTCCTTGTGCT
Keratin 35	TGCCCTGACTACCAGTCCTA	TCCAAAGCCACTCTGAACCT
Keratin 32	CATTTCAGGACCATTGAGGA	AGTCCAGTTCCCTTCCCAGA
